# Mathematical Analysis of a Fractional-Order Predator-Prey Network with Feedback Control Strategy

**DOI:** 10.1155/2021/9358881

**Published:** 2021-10-11

**Authors:** Wei Zhang, Yu Fei, Zhouhong Li, Chengdai Huang

**Affiliations:** ^1^Department of Mathematics, Yuxi Normal University, Yuxi, Yunnan 653100, China; ^2^School of Statistics and Mathematics, Yunnan University of Finance and Economics, Kunming, Yunnan 650021, China; ^3^School of Mathematics and Statistics, Xinyang Normal University, Xinyang 464000, China

## Abstract

This paper examines the bifurcation control problem of a class of delayed fractional-order predator-prey models in accordance with an enhancing feedback controller. Firstly, the bifurcation points of the devised model are precisely figured out via theoretical derivation taking time delay as a bifurcation parameter. Secondly, a set comparative analysis on the influence of bifurcation control is numerically studied containing enhancing feedback, dislocated feedback, and eliminating feedback approaches. It can be seen that the stability performance of the proposed model can be immensely heightened by the enhancing feedback approach. At the end, a numerical example is given to illustrate the feasibility of the theoretical results.

## 1. Introduction

The predator-prey model is a class of considerable models in the sphere of ecological models. The prey-predator model is one of the basic topics in ecology as a result of the pervasive importance and existence, which constitutes the convoluted food chains and food networks. The original predator-prey system was formulated in [1, 2]. It is generally accepted that time delays have been merged into biological models to elaborately delineate the authentic dynamical predator-prey use taking into account the time required for resource regeneration time, maturation period, reaction time, feeding time, and gestation period [3, 4]. Many excellent results have been attained on the study of predator-prey models [5–7].

Fractional calculus is merged into complicated, dynamical systems which extremely renovate the theory of the design and control performance for complex systems. Scholars discovered that physical phenomena in nature can be depicted more accurately by fractional-order models in comparison with classical integer-order ones [8]. Recently, quite a few researchers introduced fractional calculus into the predator-prey model and constructed fractional predator-prey models, for example, design and control of various ecological models [9–11], secure communication [12, 13], system control [14, 15], and so on. Furthermore, modelling and control based on the theory of the fractional calculus of complex systems can greatly enhance the capability of discrimination, design, and control for dynamic models since fractional calculus possesses infinite memory and more degrees of freedom [16]. Consequently, a number of available theories and principles have been further renovated on the basis of fractional calculus. Modelling and control for fractional order dynamical models have recently grown a hot research issue [17–22]. In fact, lots of biological models exhibit fractional dynamics thanks to possessing memory effects. Lately, fractional calculus has been successfully introduced into predator-prey models, and some interesting phenomena have been studied. In [23], the authors consider a fractional order delayed predator-prey system with harvesting terms. In [24], Mondal et al. found that the solutions of the fractional-order predator-prey system converge to the respective equilibrium more slowly as fractional order decreases. In [25], Chinnathambi and Rihan proposed that fractional order can strengthen the stability of the prey-predator system and hamper the occurrence of oscillation behaviors. Fractional dynamics of the predator-prey model without delays have been captured [26, 27].

The Hopf bifurcation has been widely researched for nonlinear systems, and a number of significant results have been obtained [28–30]. It should be pointed out that bifurcation problems of conventional delayed integer-order models have been fully studied, due to the maturity of theoretical methods. Nevertheless, the bifurcations of fractional-order dynamical systems have been analyzed in many papers [31–34]. In 2018, the authors gave attention to the bifurcation problems of the delayed generalized fractional-order prey-predator model with interspecific competition, and exact bifurcation results were obtained [32]. In [34], the bifurcation of a class of quaternion-valued fractional-order neural networks with time-varying delay was given, and it indicated that an increase in orders of fractional-order models will result in advancing the onset of bifurcation. In recent years, the problem of bifurcation control of fractional models with time-varying delay has attracted people's attention [35–38]. In [37], research has found that the bifurcation phenomena can be controlled by adjusting extended feedback delay or fractional order. In [38], a newly invented fractional-order PD scheme involving variable order was proposed to control the generation of bifurcation for integer-order small-world network, and innate bifurcation can be efficiently unperturbed if dedicated control gains are set.

The performance of nonlinear systems can be elevated utilizing bifurcation control methods. Recently, bifurcation control strategies have been used to delay the onset of bifurcation in fractional-order systems with time delay [39–42], such as dislocated feedback control, speed feedback control, and enhancing feedback control [43, 44]. In [43], the author considered that the feedback coefficients were smaller than the ones of ordinary feedback control in controlling the hyperchaotic Lorenz system. In [44], it was revealed that the hyperchaotic Lorenz system can be efficiently controlled based on the enhancing feedback control approach in comparison with the addressed feedback ones by selecting relatively simple external inputs and small necessary feedback coefficient. Actually, it is difficult to completely control the dynamical properties of a complex system relying on a unique feedback variable. In this instance, a larger feedback gain needs to be selected for procuring the anticipated dynamical behaviors of a nonlinear system. Hence, to acquire high-quality performance of the devised fractional-order dynamical systems, it is essential to adopt the enhancing feedback approach to control the onset of bifurcation. Up to now, few results with respect to the bifurcation control of fractional order predator-prey systems with delays by adopting the enhancing feedback control method have been obtained.

Based on the above motivations, so far, to the best of the authors' knowledge, no work has concerned the Hopf bifurcation control for a delayed fractional-order predator-prey model with feedback control. The main contributions can be summarized in three aspects:

1. An improved fractional-order predator-prey model is constructed by introducing an enhancing feedback control strategy.
2. A larger feedback gain is selected for controlling the onset of bifurcation of the created system through dislocated feedback scheme.
3. It detects that the control effects of the proposed system can be largely hoisted by using enhancing feedback approach than dislocated feedback one. This implies that the devised enhancing feedback method can attenuate the control amount compared with dislocated feedback ones. It is pointed out that the devised enhancing feedback method can attenuate the control amount compared with dislocated feedback ones.

The organization of this paper is as follows. In Section 2, several useful definitions and lemmas of fractional-order calculus are recalled. In Section 3, the discussed system is proposed. In Section 4, by analyzing the associated characteristic equation, the existence of the Hopf bifurcation of the delayed fractional-order predator-prey model with feedback control is established. In Section 5, one illustrative example is provided to demonstrate the theoretical results.  Section 6 is the conclusion of the paper.

## 2. Preliminaries

In this section, let us recall some definitions and lemmas of fractional derivatives, which can be used in the proofs of main result of Section 4.

**Definition 1 (see [16]).**

The Caputo fractional-order derivative can be defined as

(1) $$\begin{array}{c} D_{t}^{q}f\left(t\right)=\frac{1}{\Gamma \left(l-q\right)}\int_{t_{0}}^{t}{\left(t-s\right)}^{l-q-1}f^{\left(l\right)}\left(s\right)\text{d}s, \end{array}$$

where *t* ≥ *t*_0_, *l* − 1 ≤ *q* < *l* ∈ *Z*^+^, Γ(·) is the gamma function, and Γ(*s*)=∫_0_^*∞*^*t*^*s*−1^*e*^−*t*^d*t*.

By adopting Laplace transform, it follows from the Caputo fractional-order derivatives that

(2) $$\begin{array}{c} L\left\{D_{t}^{q}f\left(t\right);s\right\}=s^{q}F\left(s\right)-\sum_{k=0}^{l-1}s^{q-k-1}f^{\left(k\right)}\left(0\right),\;l-1\leq q<l\in Z^{+}. \end{array}$$

If *f*^(*k*)^(0)=0, *k*=1,2,…, *n*, then *L*{*D*_*t*_^*q*^*f*(*t*); *s*}=*s*^*q*^*F*(*s*).

**Lemma 1 (see [45]).**

The following *n*-dimensional linear fractional-order model is explored:

(3) $$\begin{array}{c} \left\{\begin{array}{c} D^{q_{1}}l_{1}\left(t\right)=a_{11}l_{1}\left(t\right)+a_{12}l_{2}\left(t\right)+\cdots +a_{1n}l_{n}\left(t\right),\\ D^{q_{2}}l_{2}\left(t\right)=a_{21}l_{1}\left(t\right)+a_{22}l_{2}\left(t\right)+\cdots +a_{2n}l_{n}\left(t\right),\\ \vdots \\ D^{q_{n}}l_{n}\left(t\right)=a_{n1}l_{1}\left(t\right)+a_{n2}l_{2}\left(t\right)+\cdots +a_{nn}l_{n}\left(t\right), \end{array}\right) \end{array}$$

where 0 < *q*_*i*_ < 1 (*i*=1,2,…, *n*). Assume that *q*_*i*_ is the lowest common multiple of the denominators *ψ*_*i*_ of *q*_*i*_, where *q*_*i*_=(*φ*_*i*_/*ψ*_*i*_), (*φ*_*i*_, *ψ*_*i*_)=1, *φ*_*i*_, *ψ*_*i*_ ∈ *Z*^+^, ∀*i*=1,2,…, *n*. It is represented as

(4) $$\begin{array}{c} \Delta \left(\lambda \right)=\left[\begin{array}{cccc} \lambda^{a_{1}}-a_{11} & -a_{12} & \cdots & -a_{1n}\\ -a_{21} & \lambda^{a_{2}}-a_{22} & \cdots & -a_{2n}\\ \vdots & \vdots & \ddots & \vdots \\ -a_{n1} & -a_{n2} & \cdots & \lambda^{a_{n}}-a_{nn} \end{array}\right]. \end{array}$$

Then, the zero solution of model (3) is globally asymptotically stable in the Lyapunov sense if all roots *λ* of the equation det(Δ(*λ*))=0 satisfy |arg(*λ*)| > (*q*_*i*_*π*/2).

**Lemma 2 (see [45]).**

The following *n*-dimensional linear fractional-order model with delays is examined:

(5) $$\begin{array}{c} \left\{\begin{array}{c} D^{q_{1}}l_{1}\left(t\right)=a_{11}l_{1}\left(t-\tau_{11}\right)+a_{12}l_{2}\left(t-\tau_{12}\right)+\cdots +a_{1n}l_{n}\left(t-\tau_{1n}\right),\\ D^{q_{2}}l_{2}\left(t\right)=a_{21}l_{1}\left(t-\tau_{21}\right)+a_{22}l_{2}\left(t-\tau_{22}\right)+\cdots +a_{2n}l_{n}\left(t-\tau_{2n}\right),\\ \vdots \\ D^{q_{n}}l_{n}\left(t\right)=a_{n1}l_{1}\left(t-\tau_{n1}\right)+a_{n2}l_{2}\left(t-\tau_{n2}\right)+\cdots +a_{nn}l_{n}\left(t-\tau_{nn}\right), \end{array}\right) \end{array}$$

where *q*_*i*_ ∈ (0,1) (*i*=1,2,…, *n*), the initial values *V*_*i*_(*t*)=Ψ_*i*_(*t*) are given for −max_*i*,*j*_, and *τ*_*i*,*j*_=−max_*i*,*j*_ ≤ *t* ≤ 0 ∀ *i*=1,2,…, *n*. For system (5), time-delay matrix *τ*=(*τ*_*i*,*j*_) ∈ (*R*^+^)_*n*×*n*_, coefficient matrix *H*=(*a*_*i*,*j*_)_*n*×*n*_, state variables *l*_*i*_(*t*), *l*_*i*_(*t* − *τ*_*i*,*j*_) ∈ *R*, and initial values Ψ_*i*_(*t*) ∈ *C*^0^[−*τ*_max_, 0]. Its fractional order is defined as *q*=(*l*_1_, *l*_2_,…, *l*_*n*_). It is defined as

(6) $$\begin{array}{c} \Delta \left(s\right)=\left[\begin{array}{cccc} s^{q_{1}}-a_{11}e^{-s\tau_{11}} & -a_{12}e^{-s\tau_{12}} & \cdots & -a_{1n}e^{-s\tau_{1n}}\\ -a_{21}e^{-s\tau_{21}} & s^{q_{2}}-a_{22}e^{-s\tau_{22}} & \cdots & -a_{2n}e^{-s\tau_{2n}}\\ \vdots & \vdots & \ddots & \vdots \\ -a_{n1}e^{-s\tau_{n1}} & -a_{n2}e^{-s\tau_{n2}} & \cdots & s^{q_{n}}-a_{nn}e^{-s\tau_{nn}} \end{array}\right]. \end{array}$$

## 3. Mathematical Model Description

A delayed ratio-dependent fractional-order predator-prey system is proposed in this paper. The mathematical model is depicted by

(7) $$\begin{array}{c} \left\{\begin{array}{c} D^{u}y_{1}\left(t\right)=y_{1}\left(t-\tau \right)\left[1-y_{1}\left(t-\tau \right)\right]-\frac{y_{1}\left(t-\tau \right)y_{2}\left(t\right)}{y_{1}\left(t-\tau \right)+\alpha y_{2}\left(t\right)},\\ D^{u}y_{2}\left(t\right)=\beta y_{2}\left(t-\tau \right)\left[\delta -\frac{y_{2}\left(t-\tau \right)}{y_{1}\left(t-\tau \right)}\right], \end{array}\right) \end{array}$$

where the relative variables and parameters of systems (7) are interpreted in Table 1.

In this paper, we consider the delayed fractional-order version of (7) which is given as follows:

(8) $$\begin{array}{c} \left\{\begin{array}{c} D^{u}y_{1}\left(t\right)=y_{1}\left(t-\tau \right)\left[1-y_{1}\left(t-\tau \right)\right]-\frac{y_{1}\left(t-\tau \right)y_{2}\left(t\right)}{y_{1}\left(t-\tau \right)+\alpha y_{2}\left(t\right)}+\phi_{1}\left[\left(y_{1}\left(t\right)\right.-y_{1}^{*}\right],\\ D^{u}y_{2}\left(t\right)=\beta y_{2}\left(t-\tau \right)\left[\delta -\frac{y_{2}\left(t-\tau \right)}{y_{1}\left(t-\tau \right)}\right]+\phi_{2}\left[\left(y_{2}\left(t\right)\right)-y_{2}^{*}\right], \end{array}\right. \end{array}$$

where *ϕ*_1_, *ϕ*_2_ represent the feedback control gains.

**Remark 1 .**

Hence, we have the observation that model (8) degenerates into the integer-order model in [46] when selecting *u*=1, *ϕ*_1_=*ϕ*_2_=0. If *ϕ*_1_=0, *ϕ*_2_ ≠ 0 or *ϕ*_1_ ≠ 0, *ϕ*_2_=0, then model (8) develops into dislocated time-delayed feedback control model. If *ϕ*_1_ ≠ 0, *ϕ*_2_ ≠ 0, then model (8) develops into the enhancing time-delayed feedback control model.

Under the condition 1+*αδ* > *δ*, model (7) occupies a unique positive equilibrium point *E*^*∗*^=(*y*_1_^*∗*^, *y*_2_^*∗*^), which complies with the following equations:

(9) $$\begin{array}{c} \left\{\begin{array}{c} 1-y_{1}^{*}-\frac{y_{2}^{*}}{y_{1}^{*}+\alpha y_{2}^{*}}=0,\\ \beta \left(\delta -\frac{y_{2}^{*}}{y_{1}^{*}}\right)=0. \end{array}\right) \end{array}$$

This means that *y*_1_^*∗*^=((1+*αδ* − *δ*)/(1+*αδ*)), *y*_2_^*∗*^=(*δ*(1+*αδ* − *δ*)/(1+*αδ*)).

**Remark 2 .**

*E* ^*∗*^ of system (7) is consistent with system (8), which does not rely on the values of control parameters *ϕ*_1_ and *ϕ*_2_=0. This implies that *E*^*∗*^ is immutable in the devised controllers.

To capture the brilliant control effects, the following essential assumption is presented in this paper:

 
(**H**1) *ϕ*_1_ ≤ 0, *ϕ*_2_ ≤ 0.

According to [45], this paper is devoted to finding out the conditions of bifurcation for model (8) using time delay as a bifurcation parameter. Then, quite a few comparative studies on bifurcation control are carried out. It can be seen that the stability performance of the controlled model can be excessively ameliorated on the basis of enhancing feedback control in comparison with the dislocated feedback control.

## 4. Main Results

In this section, time delay shall be selected as a bifurcation parameter to investigate the problem of bifurcation control for the predator-prey model (8) by utilizing enhancing feedback approach. The existence bifurcation and bifurcation point for the proposed model shall be determined.

Performing transformations *𝒲*_1_(*t*)=*y*_1_(*t*) − *y*_1_^*∗*^, *𝒲*_2_(*t*)=*y*_2_(*t*) − *y*_2_^*∗*^, then system (8) can be transformed into the following form:

(10) $$\begin{array}{c} \left\{\begin{array}{c} D^{u}W_{1}\left(t\right)=\left[W_{1}\left(t-\tau \right)+y_{1}^{*}\right]\left[1-\left(W_{1}\left(t-\tau \right)+y_{1}^{*}\right)\right]-\frac{\left(W_{1}\left(t-\tau \right)+x_{1}^{*}\right)\left(u_{2}\left(t\right)+y_{2}^{*}\right)}{W_{1}\left(t-\tau \right)+\alpha W_{2}\left(t\right)}+\phi_{1}W_{1}\left(t\right),\\ D^{u}W_{2}\left(t\right)=\beta \left(W_{2}\left(t-\tau \right)+y_{2}^{*}\right)\left[\delta -\frac{\left(W_{2}\left(t-\tau \right)+y_{2}^{*}\right)}{\left(W_{1}\left(t-\tau \right)+y_{1}^{*}\right)}\right]+\phi_{2}W_{2}\left(t\right). \end{array}\right) \end{array}$$

It can be seen from system (10) that the linearized form is

(11) $$\begin{array}{c} \left\{\begin{array}{c} D^{u}W_{1}\left(t\right)=\epsilon_{11}W_{1}\left(t-\tau \right)+\phi_{1}W_{1}\left(t\right)+\epsilon_{12}W_{2}\left(t\right),\\ D^{u}W_{2}\left(t\right)=\epsilon_{21}W_{1}\left(t-\tau \right)+\phi_{2}W_{2}\left(t\right)+\epsilon_{22}W_{2}\left(t-\tau \right), \end{array}\right) \end{array}$$

where

(12) $$\begin{array}{c} \epsilon_{11}=1-2y_{1}^{*}-\frac{y_{2}^{*}}{y_{1}^{*}+\alpha y_{2}^{*}}+\frac{y_{2}^{*}y_{1}^{*}}{{\left(y_{1}^{*}+\alpha y_{2}^{*}\right)}^{2}},\\ \epsilon_{12}=-\frac{y_{1}^{*}}{y_{1}^{*}+\alpha y_{2}^{*}}+\frac{\alpha y_{2}^{*}y_{1}^{*}}{{\left(y_{1}^{*}+\alpha y_{2}^{*}\right)}^{2}},\\ \epsilon_{21}=\frac{\beta y_{2}^{*}}{{\left(y_{1}^{*}\right)}^{2}},\\ \epsilon_{22}=\beta \delta -\frac{2\beta y_{2}^{*}}{{\left(y_{1}^{*}\right)}^{2}}. \end{array}$$

The associated characteristic equation of (11) is

(13) $$\begin{array}{c} \gamma_{1}\left(s\right)+\gamma_{2}\left(s\right)e^{-s\tau }+\gamma_{3}\left(s\right)e^{-2s\tau }=0, \end{array}$$

where

(14) $$\begin{array}{c} \gamma_{1}\left(s\right)=s^{2u}-\left(\phi_{1}+\phi_{2}\right)s^{u}+\phi_{1}\phi_{2},\\ \gamma_{2}\left(s\right)=-\left[\left(\epsilon_{11}+\epsilon_{22}\right)s^{u}-\phi_{1}\epsilon_{22}-\phi_{2}\epsilon_{11}+\epsilon_{12}\epsilon_{21}\right],\\ \gamma_{3}\left(s\right)=\epsilon_{11}\epsilon_{22}. \end{array}$$

The real and imaginary parts of *γ*_*q*_(*s*) (*q*=1,2,3) are labeled by *γ*_*q*_^*r*^, *γ*_*q*_^*i*^. Then, we obtain

(15) $$\begin{array}{c} \gamma_{1}^{r}=\varpi^{2u}\cos u\pi -\left(\phi_{1}+\phi_{2}\right)\varpi^{u}\cos\frac{u\pi }{2}+\phi_{1}\phi_{2},\\ \gamma_{1}^{i}=\varpi^{2u}\sin u\pi -\left(\phi_{1}+\phi_{2}\right)w^{u}\sin\frac{u\pi }{2},\\ \gamma_{2}^{r}=-\left[\left(\epsilon_{11}+\epsilon_{22}\right)\varpi^{u}\cos\frac{u\pi }{2}-\phi_{1}\epsilon_{22}-\phi_{2}\epsilon_{11}+\epsilon_{12}\epsilon_{21}\right],\\ \gamma_{2}^{i}=-\left(\epsilon_{11}+\epsilon_{22}\right)\varpi^{u}\sin\frac{u\pi }{2},\\ \gamma_{3}^{r}=\epsilon_{11}\epsilon_{22},\\ \gamma_{3}^{i}=0. \end{array}$$

Both sides of equation (13) are multiplied by *e*^*sτ*^; then, it follows that

(16) $$\begin{array}{c} \gamma_{1}\left(s\right)e^{s\tau }+\gamma_{2}\left(s\right)+\gamma_{3}\left(s\right)e^{-s\tau }=0. \end{array}$$

Assume that *s*=*ϖ*(cos(*π*/2)+*i*  sin(*π*/2)) (*ϖ* > 0) is a purely imaginary root of equation (16); then, it results in

(17) $$\begin{array}{c} \left\{\begin{array}{c} \left(\gamma_{1}^{r}+\gamma_{3}^{r}\right)\cos\varpi \tau -\gamma_{1}^{i}\cos\varpi \tau =-\gamma_{2}^{r},\\ \gamma_{1}^{i}\cos\varpi \tau +\left(\gamma_{1}^{r}-\gamma_{3}^{r}\right)\cos\varpi \tau =-\gamma_{2}^{i}. \end{array}\right) \end{array}$$

It is further labeled as

(18) $$\begin{array}{c} \psi_{1}\left(\varpi \right)=-\gamma_{2}^{r}\left(\gamma_{1}^{r}-\gamma_{3}^{r}\right)-\gamma_{1}^{i}\gamma_{2}^{i},\\ \psi_{2}\left(\varpi \right)=-\gamma_{2}^{i}\left(\gamma_{1}^{r}+\gamma_{3}^{r}\right)+\gamma_{1}^{i}\gamma_{2}^{r},\\ \psi_{3}\left(\varpi \right)={\left(\gamma_{1}^{r}\right)}^{2}+{\left(\gamma_{1}^{i}\right)}^{2}-{\left(\gamma_{3}^{r}\right)}^{2},\\ \rho_{1}=-\left(\phi_{1}+\phi_{2}\right),\\ \rho_{2}=\phi_{1}\phi_{2},\\ \rho_{3}=\epsilon_{11}+\epsilon_{22},\\ \rho_{4}=-\phi_{1}\epsilon_{22}-\phi_{2}\epsilon_{11}+\epsilon_{12}\epsilon_{21},\\ \rho_{5}=\epsilon_{11}\epsilon_{22}. \end{array}$$

From equation (17), it is concluded that

(19) $$\begin{array}{c} \left\{\begin{array}{c} \cos\varpi \tau =\frac{\psi_{1}\left(\varpi \right)}{\psi_{3}\left(\varpi \right)},\\ \sin\varpi \tau =\frac{\psi_{2}\left(\varpi \right)}{\psi_{3}\left(\varpi \right)}. \end{array}\right) \end{array}$$

By means of equation (19), we obtain

(20) $$\begin{array}{c} \psi_{3}\left(\varpi \right)=\psi_{1}^{2}\left(\varpi \right)+\psi_{2}^{2}\left(\varpi \right). \end{array}$$

It can be defined from equation (20) that

(21) $$\begin{array}{c} \varphi \left(\varpi \right)=\psi_{3}^{2}\left(\varpi \right)-\psi_{1}^{2}\left(\varpi \right)-\psi_{2}^{2}\left(\varpi \right)=0. \end{array}$$

In terms of equation (21), we obtain

(22) $$\begin{array}{c} \varphi \left(\varpi \right)=\varpi^{8u}+\eta_{1}\varpi^{7u}+\eta_{2}\varpi^{6u}+\eta_{3}\varpi^{5u}+\eta_{4}\varpi^{4u}+\eta_{5}\varpi^{3u}+\eta_{6}\varpi^{2u}+\eta_{7}\varpi^{u}+\eta_{8}=0, \end{array}$$

where *η*_*i*_ (*i*=1,2,…, 8) are computed in Appendix.

We further give the additional assumption:

 
(**H**2) Equation (22) has at least positive real roots.

It follows from the first equation of equation (19) that

(23) $$\begin{array}{c} \tau^{\left(k\right)}=\frac{1}{\varpi }\left[\arccos\frac{\psi_{1}\left(\varpi \right)}{\psi_{3}\left(\varpi \right)}+2k\pi \right],\;k=0,1,2,\ldots . \end{array}$$

Define the bifurcation point

(24) $$\begin{array}{c} \tau_{0}=\min\left\{\tau^{\left(k\right)}\right\},\;k=0,1,2,\ldots , \end{array}$$

where *τ*^(*k*)^ is defined by equation (23).

In the following, we will consider the stability of model (10) when *τ*=0. If *τ* is removed, the characteristic equation (16) develops into

(25) $$\begin{array}{c} s^{2u}+\varphi_{1}s^{u}+\varphi_{2}=0, \end{array}$$

where

(26) $$\begin{array}{c} \varphi_{1}=-\left(\phi_{11}+\phi_{22}+\epsilon_{11}+\epsilon_{22}\right),\\ \varphi_{2}=\phi_{1}\phi_{2}+\epsilon_{11}\epsilon_{22}+\phi_{1}\epsilon_{22}+\phi_{2}\epsilon_{11}-\epsilon_{12}\epsilon_{21}. \end{array}$$

It is obvious from *φ*_1_ > 0, *φ*_2_ > 0 that the two roots of equation (25) have negative parts satisfying Lemma 1. Thus, the positive equilibrium of fractional-order model (8) is asymptotically stable.

To obtain the conditions of bifurcation, we further assume the following:

(27) $$\begin{array}{c} \left(H3\right)\;\frac{M_{1}N_{1}+M_{2}N_{2}}{N_{1}^{2}+N_{2}^{2}}\neq 0, \end{array}$$

where *M*_1_, *M*_2_, *N*_1_, *N*_2_ are described by equation (32).

**Lemma 3 .**

Let *s*(*τ*)=*ξ*(*τ*)+*iϖ*(*τ*) be the root of equation (13) near *τ*=*τ*_*j*_ satisfying *ξ*(*τ*_*j*_)=0, *ϖ*(*τ*_*j*_)=*ϖ*_0_; then, the following transversality condition holds:

(28) Redsdτϖ=ϖ0,τ=τ0≠0,

where *ϖ*_0_, *τ*_0_ represent the critical frequency and bifurcation point of model (8).

**Proof**

The real and imaginary parts of *ℓ*_*p*_′(*s*),  *p*=1,2,3 are labeled by *ℓ*_*p*_^′*r*^, *ℓ*_*p*_^′*i*^. Using implicit function theorem to differentiate (13) concerning *τ*, the following equation can be concluded:

(29) $$\begin{array}{c} \gamma_{1}^{\prime }\left(s\right)\frac{\text{d}s}{\text{d}\tau }+\left[\gamma_{2}^{\prime }\left(s\right)\frac{\text{d}s}{\text{d}\tau }e^{-s\tau }+\gamma_{2}\left(s\right)e^{-s\tau }\left(-\tau \frac{\text{d}s}{\text{d}\tau }-s\right)\right]+\left[\gamma_{3}^{\prime }\left(s\right)\frac{\text{d}s}{\text{d}\tau }e^{-2s\tau }+\gamma_{3}\left(s\right)e^{-2s\tau }\left(-2\tau \frac{\text{d}s}{\text{d}\tau }-s\right)\right]=0. \end{array}$$

Equation (13) implies that *γ*_3_′(*s*)=0. Mathematically, from equation (32), we obtain

(30) $$\begin{array}{c} \frac{\text{d}s}{\text{d}\tau }=\frac{M\left(s\right)}{N\left(s\right)}, \end{array}$$

where

(31) $$\begin{array}{c} M\left(s\right)=s\left[\gamma_{2}\left(s\right)e^{-s\tau }+2\gamma_{3}\left(s\right)e^{-2s\tau }\right],\\ N\left(s\right)=\gamma_{1}^{\prime }\left(2\right)+\left[\gamma_{2}^{\prime }\left(s\right)-\tau \gamma_{2}\left(s\right)\right]e^{-s\tau }-2\tau \gamma_{3}\left(s\right)e^{-2s\tau }. \end{array}$$

From equation (30), we obtain

(32) Redsdτϖ=ϖ0,τ=τ0=M1N1+M2N2N12+N22,

where

(33) $$\begin{array}{c} M_{1}=\varpi_{0}\left(\gamma_{2}^{r}\sin\;\varpi_{0}\tau_{0}-\gamma_{2}^{i}\cos\;\varpi_{0}\tau_{0}+2\gamma_{3}^{r}\sin\;2\varpi_{0}\tau_{0}-2\gamma_{3}^{i}\cos\;2\varpi_{0}\tau_{0}\right),\\ M_{2}=\varpi_{0}\left(\gamma_{2}^{r}\cos\;\varpi_{0}\tau_{0}+\gamma_{2}^{i}\sin\;\varpi_{0}\tau_{0}+2\gamma_{3}^{r}\cos\;2\varpi_{0}\tau_{0}+2\gamma_{3}^{i}\sin\;2\varpi_{0}\tau_{0}\right),\\ N_{1}=\gamma_{1}^{\prime r}+\left(\gamma_{2}^{\prime r}-\tau_{0}\gamma_{2}^{r}\right)\cos\;\varpi_{0}\tau_{0}+\left(\gamma_{2}^{\prime i}-\tau_{0}\gamma_{2}^{i}\right)\sin\;\varpi_{0}\tau_{0}-2\tau_{0}\gamma_{3}^{r}\cos\;2\varpi_{0}\tau_{0},\\ N_{2}=\gamma_{1}^{\prime i}+\left(\gamma_{2}^{\prime i}-\tau_{0}\gamma_{2}^{i}\right)\cos\;\varpi_{0}\tau_{0}-\left(\gamma_{2}^{\prime r}-\tau_{0}\gamma_{2}^{r}\right)\sin\;2\varpi_{0}\tau_{0}+2\tau_{0}\gamma_{3}^{r}\sin\;2\varpi_{0}\tau_{0}. \end{array}$$

(**H**3) indicates that transversality condition holds, which completes the proof of Lemma 3.

Based on the previous analysis, the following theorem is obtained.

**Theorem 1 .**

Under conditions (**H**1)–(**H**3), the following results are available:

1. Equilibrium point *E*^*∗*^ of model (8) is asymptotically stable for *τ* ∈ (0, *τ*_0_).
2. Model (8) undergoes a Hopf bifurcation at *E*^*∗*^ for *τ*=*τ*_0_, that is, it has one branch of periodic solutions that can bifurcate from the zero equilibrium point at *τ*=*τ*_0_.

**Remark 3 .**

Due to the higher order of equation (22), it is not always easy to deal with all the positive real roots of it theoretically. However, it is simple to procure the concrete of these positive real roots of equation (22) with the help of Maple numerical software. Hence, the values of *τ*_0_ can be accurately concluded.

**Remark 4 .**

It can be seen that a small feedback gain cannot control the onset of bifurcation of a delayed fractional predator-prey model based on dislocated feedback strategy in [47]. Based on the dislocation feedback method, an extended delayed feedback method was designed to control bifurcation of a delayed fractional predator-prey model despite selecting a small feedback gain in [37]. It should be pointed out that the extended feedback delay plays an essential role in postponing Hopf bifurcation for such model. In this paper, by choosing enhancing feedback method, the bifurcation of the proposed model can be easily controlled provided that a set of smaller feedback gains is selected. Contrarily, the bifurcation of the devised model can be controlled by using the dislocated feedback approach only if a larger feedback gain is requested. It exhibits that the onset of the bifurcation for the fractional delayed predator-prey model can be delayed and satisfactory bifurcation control effects are realized compared with the dislocated feedback approaches in this paper. This shows that the devised enhancing feedback method can reduce the control cost compared with dislocated feedback ones.

**Remark 5 .**

The influence of fractional order on the bifurcation point is adequately discussed. This means that better effects in delaying the onset of bifurcation can be achieved as fractional order decreases if feedback gain is established. At the same time, it can be seen that better control effects can be gained in delaying bifurcation of the proposed model by applying the enhancing feedback method instead of dislocated feedback one.

## 5. Numerical Example

In this section, we give one example to show the feasibility and effectiveness of the results obtained in this paper. All of the simulation results are based on Adama-Bashforth-Moulton predictor-corrector scheme. In this section, numerical simulations are presented. For the purpose of comparison, the parameters were identically derived from [46]. *E*^*∗*^ can be obtained as (*y*_1_^*∗*^, *y*_2_^*∗*^)=(0.5775, 0.3465). The initial values are all designated as (*y*_1_(0), *y*_2_(0))=(0.5, 0.3). Investigate the controlled model

(34) $$\begin{array}{c} \left\{\begin{array}{c} D^{u}y_{1}\left(t\right)=y_{1}\left(t-\tau \right)\left[1-y_{1}\left(t-\tau \right)\right]-\frac{y_{1}\left(t-\tau \right)y_{2}\left(t\right)}{y_{1}\left(t-\tau \right)+\alpha y_{2}\left(t\right)}+\phi_{1}\left[\left(y_{1}\left(t\right)-y_{1}^{*}\right.\right],\\ D^{u}y_{2}\left(t\right)=\beta y_{2}\left(t-\tau \right)\left[\delta -\frac{y_{2}\left(t-\tau \right)}{y_{1}\left(t-\tau \right)}\right]+\phi_{2}\left[\left(y_{2}\left(t\right)-y_{2}^{*}\right.\right], \end{array}\right) \end{array}$$

where *α*=0.7, *β*=0.9, *δ*=0.6, *y*_1_^*∗*^=0.5775, and *y*_2_^*∗*^=0.3465.

Some results are available in Table 2.

**Figure 1:**
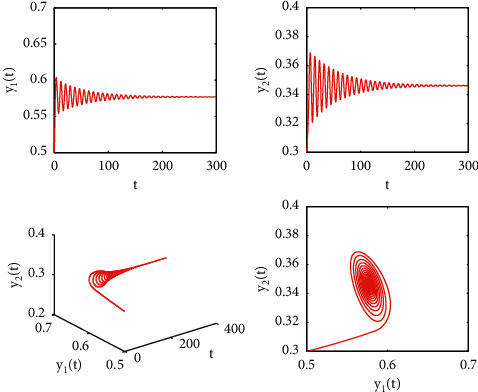
Time responses and portrait plots of model (34) with *u*=0.95, *ϕ*_1_=−0.25, *ϕ*_2_=−0.18, and *τ*=2.1 < *τ*_0_=2.2072.

**Figure 2:**
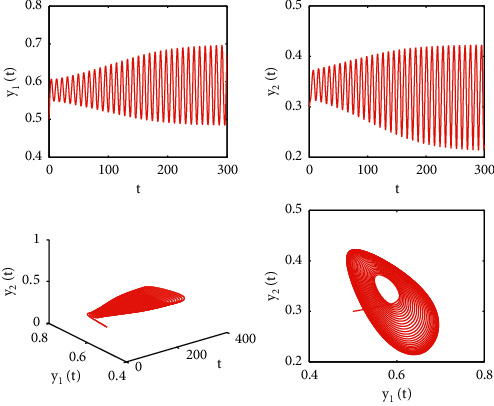
Time responses and portrait plots of model (34) with *u*=0.95, *ϕ*_1_=−0.25, *ϕ*_2_=−0.18, and *τ*=2.3 > *τ*_0_=2.0902.

**Figure 3:**
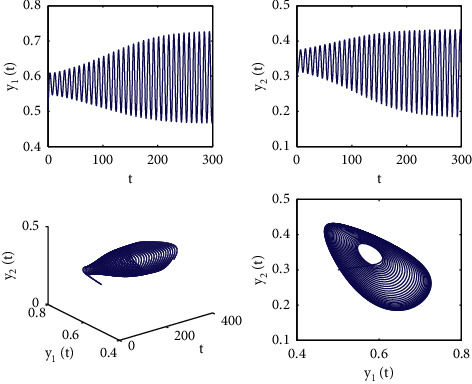
Time responses and portrait plots of model (34) with *u*=1, *ϕ*_1_=−0.25, *ϕ*_2_=−0.18, and *τ*=2.1.

**Figure 4:**
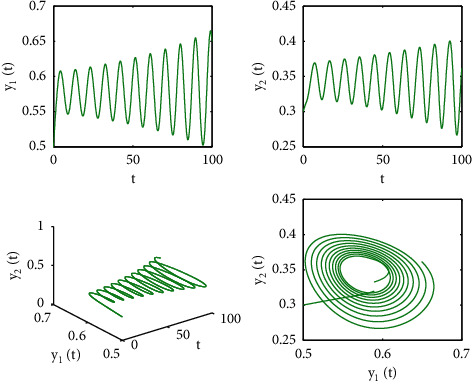
Time responses and portrait plots of model (34) with *u*=0.95, *ϕ*_1_=0, *ϕ*_2_=−0.18, and *τ*=2.1.

**Figure 5:**
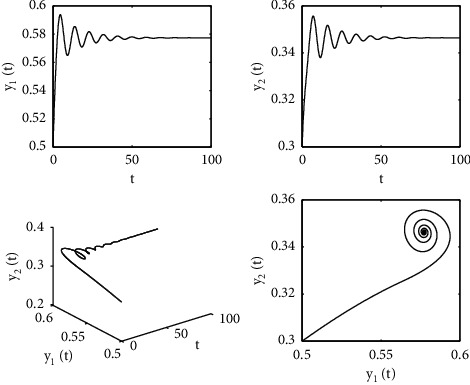
Time responses and portrait plots of model (34) with *u*=0.95, *ϕ*_1_=0, *ϕ*_2_=−0.4, and *τ*=2.1.

**Figure 6:**
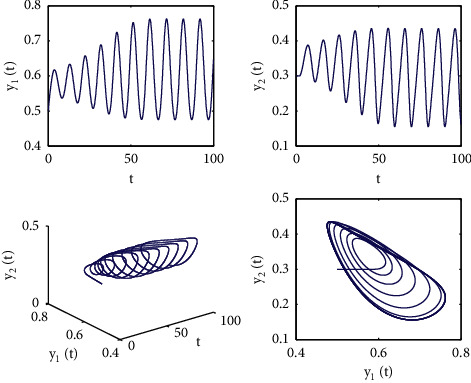
Time responses and portrait plots of model (34) with *u*=0.95, *ϕ*_1_=−0.25, *ϕ*_2_=0, and *τ*=2.1.

**Figure 7:**
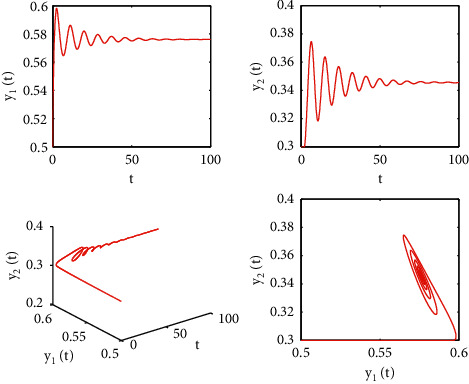
Time responses and portrait plots of model (34) with *u*=0.95, *ϕ*_1_=−1.2, *ϕ*_2_=0, and *τ*=2.1.

**Figure 8:**
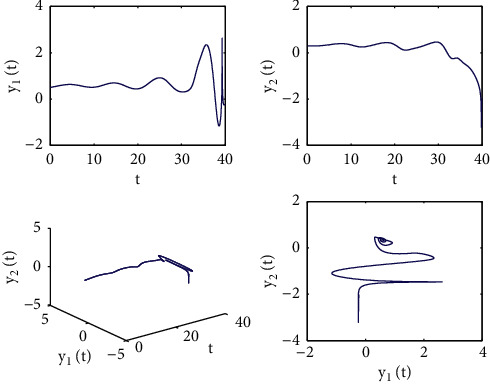
Time responses and portrait plots of model (34) with *u*=0.95, *ϕ*_1_=0, *ϕ*_2_=0, and *τ*=2.1.

Figure 1 describes the asymptotic stability of system (34) when *τ*=2.1 < *τ*_0_=2.2072. Hopf bifurcation occurs, and the instability of system (34) with *τ*=2.3 > *τ*_0_=2.2072 is depicted in Figure 2. It can be observed that system (34) turns unstable when *u*=1 is selected and other parameters are established (see Figure 3). Moreover, it can be seen from Figure 4 that system (34) becomes unstable by choosing *τ*=2.1 with dislocated control *ϕ*_1_=0, *ϕ*_2_=−0.18. Furthermore, it can be seen from Figure 5 that system (34) converges to *E*^*∗*^ when choosing larger *ϕ*_2_=−0.4. This means that the onset of bifurcation of system (34) can be controlled by taking larger feedback gain in terms of dislocated control. Similar phenomena can be observed in Figures 6 and 7. When abolishing the controllers, that is, *ϕ*_1_=0, *ϕ*_2_=0, it is clear that system (34) becomes unstable, which is illustrated in Figure 8.

**Figure 9:**
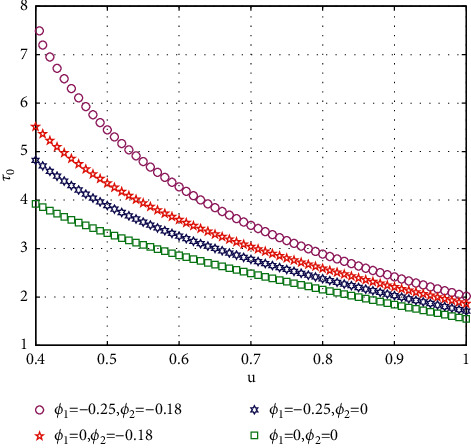
Comparison of the values of *τ*_0_ versus *u* for model (34).

**Figure 10:**
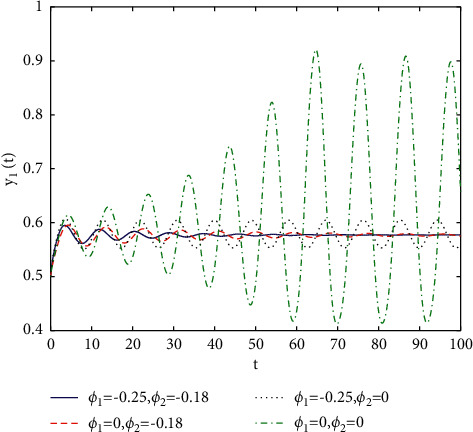
Time responses of model (34) with *u*=0.83 and *τ*=2.3.

**Figure 11:**
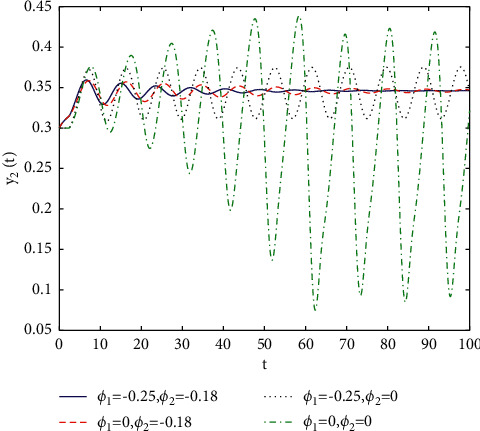
Time responses of model (34) with *u*=0.83 and *τ*=2.3.

**Figure 12:**
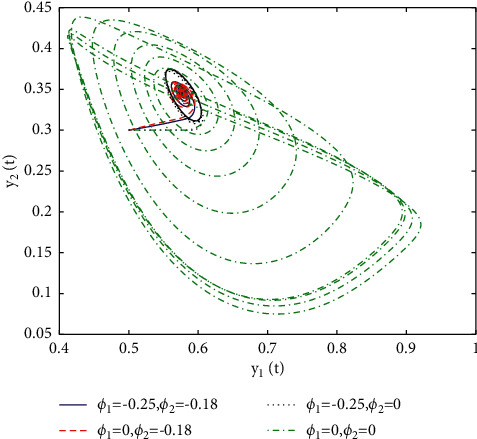
Portrait plots of model (34) with *u*=0.83 and *τ*=2.3.

**Figure 13:**
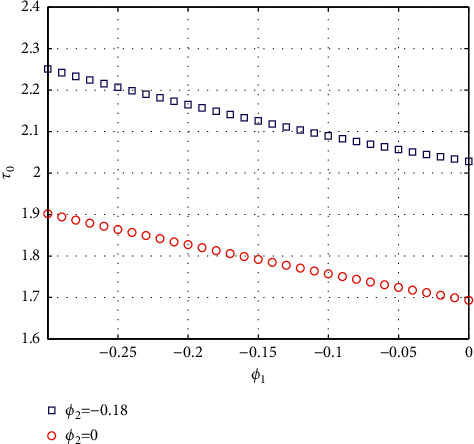
Comparison of the values of *τ*_0_ versus *ϕ*_1_ for model (34) with *u*=0.95.

**Figure 14:**
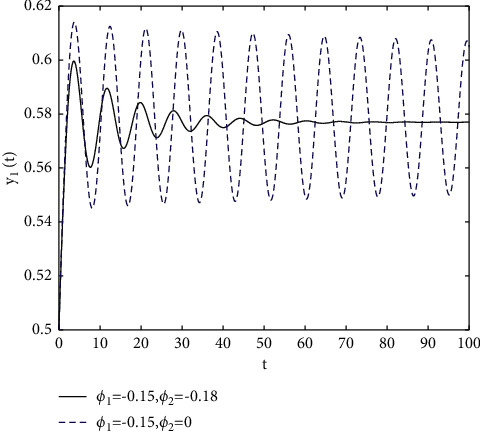
Time responses of model (34) with *u*=0.95 and *τ*=1.8.

**Figure 15:**
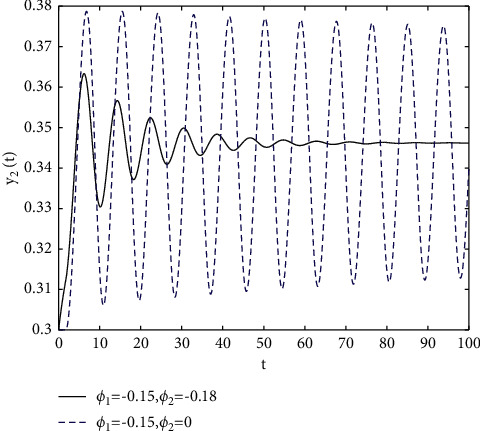
Time responses of model (34) with *u*=0.95 and *τ*=1.8.

**Figure 16:**
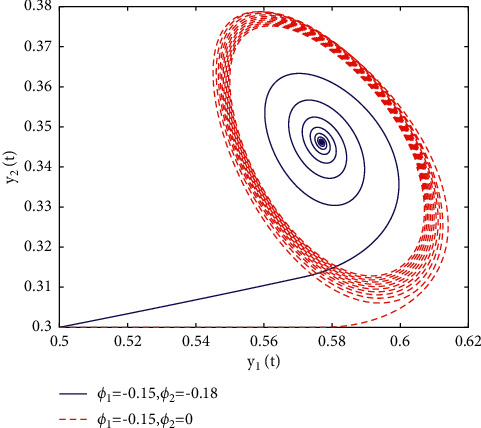
Portrait plots of model (34) with *u*=0.95 and *τ*=1.8.

**Figure 17:**
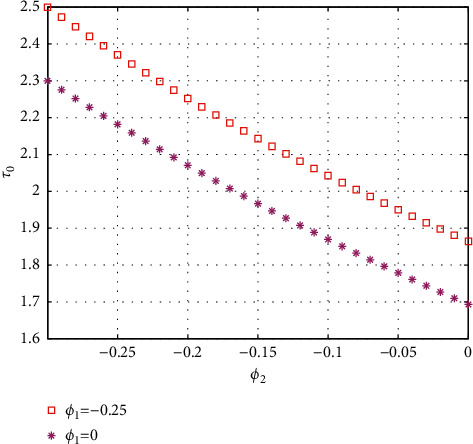
Comparison of the values of *τ*_0_ versus *ϕ*_2_ for model (34) with *ϕ*=0.95.

**Figure 18:**
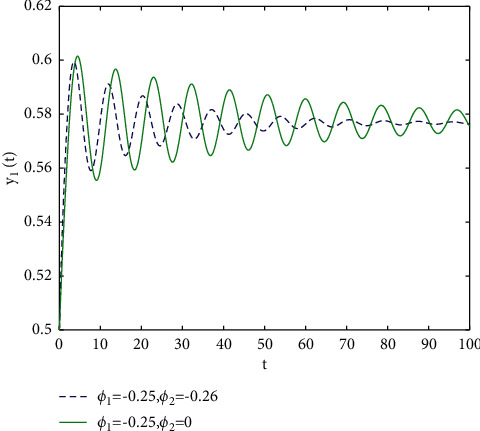
Time responses of model (34) with *u*=0.95 and *τ*=2.1.

**Figure 19:**
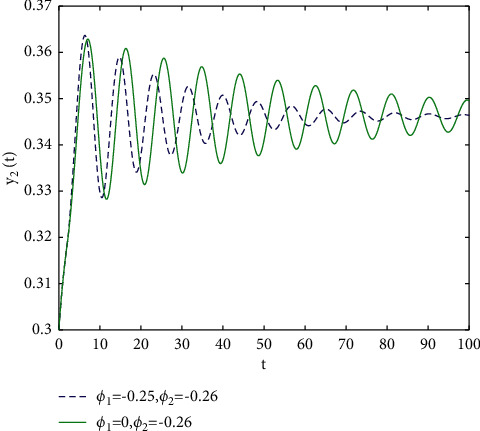
Time responses of model (34) with *u*=0.95 and *τ*=2.1.

**Figure 20:**
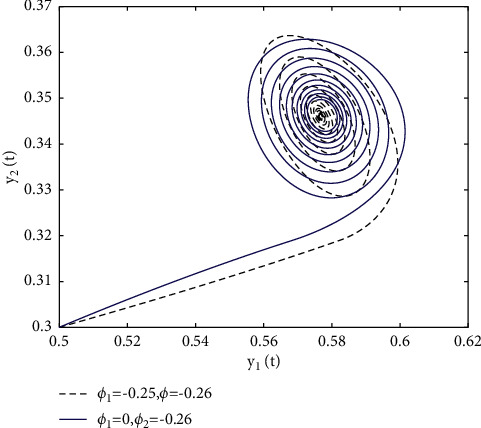
Portrait plots of model (34) with *u*=0.95 and *τ*=2.1.

In fact, by changing the values of *u*, the corresponding *τ*_0_ can be obtained by taking the enhancing feedback control and dislocated feedback control and without control approaches, respectively, which is illustrated in Figure 9. This implies that enhancing feedback control transcends others, which is verified in simulation results in Figures 10–12. When establishing *u*, *ϕ*_2_=−0.18, or *ϕ*_2_=0, the values of *τ*_0_ can be determined as *ϕ*_1_ varies, which is depicted in Figure 13. Figure 13 also means that enhancing feedback control exceeds dislocated feedback control, as shown in Figures 14–16. The order is selected as *u*=0.95 and the parameter is designated as *ϕ*_1_=−0.25, taking *ϕ*_1_=0 and varying *ϕ*_2_ in model (34), and the values of *τ*_0_ can be computed, which is described in Figure 17. It can also be seen from Figure 17 that enhancing feedback control overmatches dislocated feedback control, which is very consistent with numerical simulations in Figures 18–20.

## 6. Conclusion

In this paper, the issue of bifurcation for a delayed of fractional predator-prey model with feedback control has been given. By utilizing time delay as the bifurcation parameter, a number of criteria to ensure the existence of the Hopf bifurcation for the fractional-order predator-prey model with feedback control were studied. Mathematical analysis and simulation results further reveal that better efficiency of bifurcation control has been obtained in terms of enhancing feedback approach compared to dislocated and uncontrolled ones with partially or completely removing the branch for feedback gains. It can be seen that the onset of bifurcation can be controlled for the dislocated feedback schemes, yet greater feedback gains must be taken, which increases the cost of control model. Contrarily, the stability performance of the controlled model can be extremely ameliorated on account of the designed enhancing feedback methodology by choosing smaller feedback gains. In addition, numerical results have been provided to confirm the efficiency of the derived theoretical results.

## Figures and Tables

**Table 1:** Parameter values for mathematical model (7).

Symbols	Interpretation
*y*_1_(*t*)	Denotes the population densities of prey at time *t*
*y*_2_(*t*)	Denotes the population densities of predator at time *t*
*u*	*u* ∈ (0,1] is fractional order
*α*	Positive constant
*β*	Positive constant
*δ*	Positive constant
*τ*	Time delay for both the densities of the predator and the prey

**Table 2:** Comparative results based on different parameters and approaches of system (34).

Feedback strategy	Fractional order	Feedback gain	Critical frequency	Bifurcation point	Simulations
Enhancing control	*u*=0.95	*ϕ*_1_=−0.25, *ϕ*_2_=−0.18	*ϖ*_0_=0.7063	*τ*_0_=2.2072	Figures 1 and 2
Enhancing control	*u*=1	*ϕ*_1_=−0.25, *ϕ*_2_=−0.18	*ϖ*_0_=0.7256	*τ*_0_=2.0148	Figure 3
Dislocated control	*u*=0.95	*ϕ*_1_=0, *ϕ*_2_=−0.18	*ϖ*_0_=0.6799	*τ*_0_=2.0283	Figure 4
Dislocated control	*u*=0.95	*ϕ*_1_=0, *ϕ*_2_=−0.4	*ϖ*_0_=0.6073	*τ*_0_=2.5569	Figure 5
Dislocated control	*u*=0.95	*ϕ*_1_=−0.25, *ϕ*_2_=0	*ϖ*_0_=0.7282	*τ*_0_=1.8642	Figure 6
Dislocated control	*u*=0.95	*ϕ*_1_=−1.2, *ϕ*_2_=0	*ϖ*_0_=0.6448	*τ*_0_=2.4733	Figure 7
Without control	*u*=0.95	*ϕ*_1_=0, *ϕ*_2_=0	*ϖ*_0_=0.7086	*τ*_0_=1.6044	Figure 8

## Data Availability

No data were used to support this study.

## Appendix

(A.1)
$$\begin{array}{c} \eta_{1}=4\rho_{1}\cos\frac{u\pi }{2},\\ \eta_{2}=\left(6\rho_{1}^{2}-\rho_{3}^{2}\right)\left(\sin^{2}\;p\pi \;\sin^{2}\frac{u\pi }{2}+\;\cos^{2}\;p\pi \;\cos^{2}\frac{u\pi }{2}\right)+\left(2\rho_{1}^{2}-\rho_{3}^{2}\right)\left(\sin^{2}\;u\pi \;\cos^{2}\frac{u\pi }{2}+\;\cos^{2}\;u\pi \;\sin^{2}\frac{u\pi }{2}\right)+4\rho_{1}^{2}\sin^{2}\;u\pi \;\cos u\pi +4\rho_{2}\cos u\pi ,\\ \eta_{3}=2\left[\rho_{1}\left(\rho_{1}^{2}-\rho_{3}^{2}\right)+\left(2\rho_{1}\rho_{2}-\rho_{3}\rho_{4}\right)\sin^{2}\;u\pi +\left(6\rho_{1}\rho_{2}-\rho_{3}\rho_{4}\right)\cos^{2}\;u\pi \right]\cos\frac{u\pi }{2}\\ +8\rho_{1}\rho_{2}\sin u\pi \;\cos u\pi \;\sin\frac{u\pi }{2},\\ \eta_{4}=\rho_{1}^{2}\left(\rho_{1}^{2}-\rho_{3}^{2}\right)+\left(2\rho_{2}^{2}+4\rho_{2}\rho_{1}^{2}+2\rho_{5}\rho_{3}^{2}-2\rho_{1}\rho_{3}\rho_{4}-\rho_{4}^{2}-2\rho_{5}^{2}\right)\sin^{2}\;p\pi +\left(6\rho_{2}^{2}-\rho_{4}^{2}-2\rho_{5}^{2}\right)\cos^{2}\;u\pi \\ +4\left(\rho_{2}\rho_{1}^{2}-2\rho_{5}\rho_{3}^{2}\right)\cos u\pi \sin^{2}\frac{p\pi }{2}+2\left(\rho_{5}\rho_{3}^{2}+6\rho_{2}\rho_{1}^{2}-\rho_{2}\rho_{3}^{2}-2\rho_{1}\rho_{3}\rho_{4}\right)\cos u\pi \cos^{2}\frac{u\pi }{2},\\ \eta_{5}=2\left(2\rho_{1}\rho_{2}^{2}+2\rho_{3}\rho_{4}\rho_{5}-\rho_{1}\rho_{4}^{2}-2\rho_{1}\rho_{5}^{2}\right)\sin u\pi \;\sin\frac{p\pi }{2}+\rho_{1}\left(4\rho_{2}\rho_{1}^{2}+2\rho_{5}\rho_{3}^{2}-2\rho_{1}\rho_{3}\rho_{4}-\rho_{2}\rho_{3}^{2}\right)\cos u\pi \\ \cdot \sin^{2}\frac{u\pi }{2}+2\rho_{1}\left(\rho_{5}\rho_{3}^{2}+2\rho_{2}\rho_{1}^{2}-\rho_{1}\rho_{3}\rho_{4}-\rho_{2}\rho_{3}^{2}\right)\cos^{3}\frac{u\pi }{2}+\left(12\rho_{1}\rho_{2}^{2}+4\rho_{3}\rho_{4}\rho_{5}-4\rho_{1}\rho_{5}^{2}-2\rho_{1}\rho_{4}^{2}-4\rho_{2}\rho_{3}\rho_{4}\right)\cos p\pi \;\cos\frac{u\pi }{2},\\ \eta_{6}=\left(2\rho_{1}^{2}\rho_{2}^{2}+4\rho_{1}\rho_{3}\rho_{4}\rho_{5}-\rho_{1}^{2}\rho_{4}^{2}-\rho_{2}^{2}\rho_{3}^{2}-\rho_{3}^{2}\rho_{5}^{2}-2\rho_{1}^{2}\rho_{5}^{2}-2\rho_{2}\rho_{5}\rho_{3}^{2}\right)\sin^{2}\frac{u\pi }{2}\\ +\left(2\rho_{2}\rho_{5}\rho_{3}^{2}+4\rho_{1}\rho_{3}\rho_{4}\rho_{5}+6\rho_{1}^{2}\rho_{2}^{2}-\rho_{1}^{2}\rho_{4}^{2}-\rho_{2}^{2}\rho_{3}^{2}-\rho_{3}^{2}\rho_{5}^{2}-2\rho_{1}^{2}\rho_{5}^{2}-4\rho_{1}\rho \rho_{2}\rho_{3}\rho_{4}\right)\cos^{2}\frac{u\pi }{2}\\ +\left(4\rho_{2}^{3}+2\rho_{5}\rho_{4}^{2}-2\rho_{2}\rho_{4}^{2}-4\rho_{2}\rho_{5}^{2}\right)\cos u\pi ,\\ \eta_{7}=2\left(2\rho_{1}\rho_{2}^{3}+\rho_{1}\rho_{5}\rho_{4}^{2}+2\rho_{2}\rho_{3}\rho_{4}\rho_{5}-\rho_{1}\rho_{2}\rho_{4}^{2}-\rho_{3}\rho_{4}\rho_{5}^{2}-\rho_{3}\rho_{4}\rho_{2}^{2}-2\rho_{1}\rho_{2}\rho_{5}^{2}\right)\cos\frac{u\pi }{2},\\ \eta_{8}={\left(\rho_{2}^{2}-\rho_{5}^{2}\right)}^{2}-\rho_{4}^{2}\left(\rho_{2}^{2}+\rho_{5}^{2}\right)+2\rho_{2}\rho_{5}\rho_{4}^{2}. \end{array}$$
